# Stimulus frequency-dependent inhibition of micturition contractions of the urinary bladder by electrical stimulation of afferent Aβ, Aδ, and C fibers in cutaneous branches of the pudendal nerve

**DOI:** 10.1007/s12576-016-0468-x

**Published:** 2016-07-06

**Authors:** Akiko Onda, Sae Uchida, Harue Suzuki, Harumi Hotta

**Affiliations:** 1 Department of Autonomic Neuroscience, Tokyo Metropolitan Institute of Gerontology, 35-2 Sakaecho, Itabashi-ku, Tokyo, 173-0015 Japan; 2 Division of Rheumatology and Allergy, Department of Internal Medicine, Teikyo University School of Medicine, Tokyo, 173-8606 Japan; 3 Graduate School of the University of Human Arts and Sciences, Saitama, 339-8539 Japan

**Keywords:** Urinary bladder, Reflexes, Electrical stimulation, Myelinated nerve fibers, Unmyelinated nerve fibers

## Abstract

We aimed to examine the afferent mechanisms for the reflex inhibition of the rhythmic micturition contractions (RMCs) of the urinary bladder induced by stimulation of the perineal skin afferents in urethane-anesthetized rats. Electrical stimulation (pulse duration: 0.5 ms) was applied to the cutaneous branches of the pudendal nerve (CBPN) at frequencies of 0.1, 1, and 10 Hz for 1 min. Nerve fiber groups were defined by recording compound action potentials from CBPN. Activation of only Aβ fibers (0.2 V) produced an inhibition of RMCs at 7–11 min after the onset of stimulation (late inhibition), at any tested frequency. Additional activation of Aδ fibers (1 V) produced additional early inhibition (immediately after stimulation) at 1 and 10 Hz. Furthermore, additional activation of C fibers (10 V) at 10 Hz completely stopped RMCs for >10 min. This strong inhibition persisted after local application of capsaicin to the stimulating CBPN. We conclude that activities of Aβ, Aδ, and C afferent fibers, without capsaicin-sensitive channels, can contribute to the inhibition of bladder contractions.

## Introduction

Afferent information from perineal skin induces not only cutaneous sensation but also various autonomic responses, including ejaculation and modulation of vesical contraction. Electrical stimulation of the pudendal nerve [1–5] or natural stimulation of perineal skin [6–8] can inhibit reflex contractions of the urinary bladder induced by bladder distension in anesthetized animals. However, the types of somatic afferent fibers involved in such reflex inhibition are still to be accurately determined.

We have recently found that a gentle mechanical stimulus to the perineal skin applied by a soft roller can strongly inhibit rhythmic micturition contractions (RMCs) in anesthetized rats, and that the stimulation induces a low-frequency (range, 0.03–11 Hz) excitation of low-threshold mechanoreceptive Aβ, Aδ, and C fibers in the cutaneous branch of the pudendal nerve (CBPN) [8]. Our finding that the firing rates of cutaneous afferent fibers during rolling stimulation were <11 Hz indicates that relatively low-frequency excitation of cutaneous low-threshold mechanoreceptive fibers is sufficient to inhibit RMCs. The efficacy of low-frequency activity is in agreement with previous studies, which report that low-frequency electrical stimulation (5–10 Hz) of pudendal afferent fibers induces inhibition of the micturition reflex, whereas higher frequency stimuli (20–40 Hz) of the same afferent fibers are ineffective or have opposite effects on the bladder contractions in anesthetized cats [1, 2, 9].

We aimed to systematically study the contribution of cutaneous afferent Aβ, Aδ, and C fiber groups on the inhibition of the micturition reflex. For this purpose, vesical reflex responses to the electrical stimulation of perineal cutaneous afferent nerve fibers were determined in anesthetized rats with distended bladders. Electrical stimulation was applied to CBPN at different frequencies with three different strengths to evoke the activation of different sets of fiber groups, defined by recording afferent volleys from CBPN. Part of the study was published as an abstract form [10].

## Methods

The experiments were performed in 10 adult (4–8 months of age) male Wistar or Fischer rats. This study was conducted in accordance with the Guidelines for Proper Conduct of Animal Experiments (established by the Science Council of Japan in 2006) and was approved by the animal care and use committee of Tokyo Metropolitan Institute of Gerontology. All surgeries and data collection were performed under urethane anesthesia. Basic preparation, including dose of anesthesia, maintenance of artificial respiration and core body temperature, and recordings of intravesical pressure and blood pressure, were the same as in a previous study [8]. During data collection, gallamine triethiodide (20 mg/kg, i.v.) was administered to avoid contamination of skeletal muscle activity. A catheter inserted into the bladder via the anterior urethra was connected to a transducer (TP-200T, Nihon Kohden, Tokyo) via a T-shaped connector. The bladder was filled with saline to produce isovolumic bladder RMCs.

After cutting the skin on the left side in the prone position, CBPN, alternatively named the posterior cutaneous nerve of the thigh [11] or the scrotal nerve [12], was separated and cut at >20 mm caudal to the sacral plexus. The cavity was kept open by pulling back the edge of the cut skin with threads, and the cavity was filled with warm paraffin oil. The central cut segments of the nerve were placed on bipolar platinum–iridium wire stimulation electrodes, and repetitive rectangular pulses (0.5 ms) were delivered to the nerve. During CBPN stimulation, the evoked compound action potential of CBPN was recorded at 17–25 mm proximal from the stimulation site in five rats.

Values are expressed as mean ± standard error. Changes in RMCs induced by CBPN stimulation were assessed using repeated measures one-way analysis of variance (ANOVA), followed by Dunnett’s multiple comparison test or two-way ANOVA repeated by time. Statistical significance was set at the 5 % level. One or two trials in each rat were used for the statistical analysis.

## Results and Discussion

**Fig. 1:**
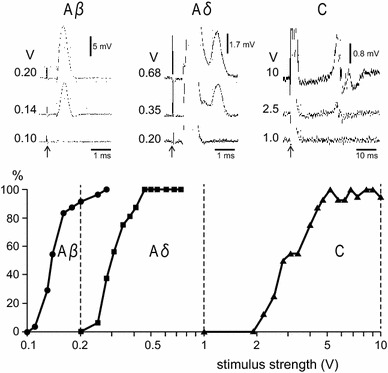
Compound action potentials of Aβ, Aδ, and C fibers induced by electrical stimulation of CBPN. Length between stimulation and recording site was 25 mm. *Upper* sample recordings of compound action potentials evoked by single electrical pulses at different stimulus intensities. *Lower* graph of the relation of stimulus strength (V) and magnitude of evoked volleys of each fiber expressed as % of maximum

The afferent fiber groups involved in the reflex inhibition of RMCs were examined by the electrical stimulation of CBPN with different stimulus intensities and frequencies for repetitive stimulation. We varied stimulus intensities as a method for preferentially activating fiber groups and confirmed our ability to selectively activate particular groups or combinations of groups by recording the evoked volleys of nerve action potentials and identifying compound action potentials with different conduction velocities (Fig. 1). As the stimulus intensity increased, three different compound action potential waves corresponding to Aβ, Aδ, and C fibers were gradually elicited, in that order. The mean threshold intensities for Aβ, Aδ, and C nerve fibers were 0.12 V (range 0.10–0.13 V; *n* = 4), 0.30 V (0.25–0.38 V), and 3.1 V (2.2–4.0 V), respectively. Evoked action potentials were identified by the maximum conduction velocities of 42.2 m/s (range 38.5–48.6 m/s) for Aβ fibers, 12.6 m/s (12.1–13.2 m/s) for Aδ fibers, and 1.2 m/s (1.1–1.3 m/s) for C fibers. We chose three stimulus intensities to study the repetitive stimulation. The stimulus intensities were set at 0.2 V (suprathreshold for Aβ fibers but subthreshold for Aδ and C fibers), 1.0 V (suprathreshold for Aβ and Aδ fibers but subthreshold for C fibers), and 10 V (suprathreshold for all fibers). CBPN were electrically stimulated with these intensities at different frequencies (0.1, 1, 10 Hz) for 1 min, and responses of RMCs to these stimuli were examined in seven rats.

**Fig. 2:**
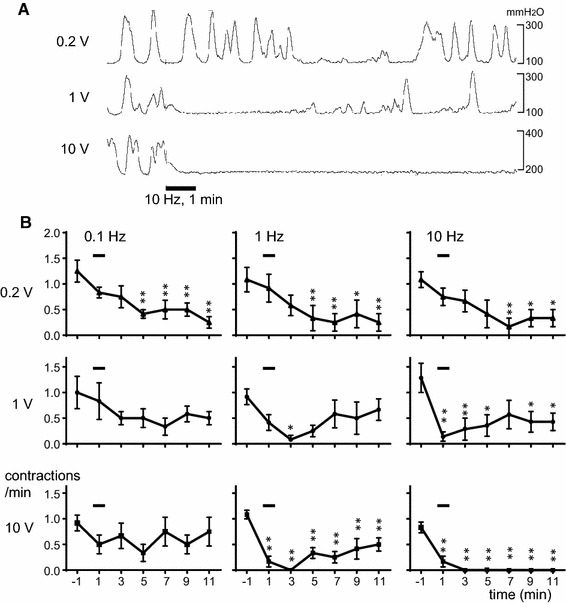
Effects of electrical stimulation of CBPN on RMCs of the urinary bladder. **a** Sample recordings of RMCs in one rat. Stimulation for 1 min at 10 Hz was applied as indicated by *bottom bar*. **b** Peristimulus time histograms of RMCs, compiled from 6 to 7 trials in seven rats. Each *point* represents mean ± standard error for mean frequency of the contractions counted every 2 min and expressed as frequencies per min. Onset of stimulation was set as time zero. *p < 0.05, **p < 0.01; significantly different from prestimulus basal values using repeated measures one-way ANOVA followed by Dunnett’s test

When the urinary bladder was infused with saline (0.8–1.5 ml), the bladder started to produce micturition contractions (basal pressure: 80–170 mm H_2_O, peak pressure: 270–660 mm H_2_O) rhythmically (frequency 0.5–3/min) (see specimen records before the stimuli in Fig. 2a). RMCs could be observed for >2 h under the resting condition without CBPN stimulation. When RMCs were stable for at least 5 min, we started to apply electrical stimulation to CBPN.

Figure 2a illustrates sample recordings in which 10 Hz stimulation of CBPN afferents for 1 min at various intensities affected RMCs in an intensity-dependent manner. The time courses of the effect of electrical stimulation of CBPN at various intensities (0.2, 1, and 10 V) and frequencies (0.1, 1, and 10 Hz) are summarized as time histograms of contraction counted every 2 min and expressed as frequencies per minute in the graphs of Fig. 2b. To make these graphs, each vesical contraction was counted as one contraction only when its amplitude was maintained above one third of the prestimulus control size according to Sato et al. [13].

Stimulation with 0.2 V produced no obvious responses in RMCs during and immediately after the end of stimulation at any frequency tested (Fig. 2b, upper row). However, frequency of RMCs gradually decreased after that, taking several minutes. Significant decrease was observed at 7–11 min after the onset of stimulation at 0.1–10 Hz. The decreased frequency of RMCs recovered within 20 min after stimulation. There were no significant differences in changes in RMCs among stimulation at 0.1, 1, and 10 Hz stimuli. Our results indicate that the activation of Aβ afferents produced late inhibition at 0.1–10 Hz. The result that Aβ fibers do not contribute to early inhibition was consistent with that of the previous study by Sato et al. [14] who focused on early effects (during stimulation) of electrical stimulation of a hind limb nerve. A delayed inhibition of bladder contractions similar to our result was reported by the stimulation of the L6 spinal nerve using motor threshold pulses [15]. However, they reported that the effect was only observed at 10 Hz of stimulus frequency, but not at 1 or 20 Hz; in contrast, our stimulation at much lower frequencies (0.1–1 Hz) was sufficient to cause the same effects as 10 Hz stimulation. The L6 spinal nerve contains not only perineal skin afferents but also afferent fibers from other skin areas, muscles, joints, and visceral organs (e.g., the urinary bladder). Therefore, the difference may be caused by an additional activation of the other afferent fibers by the stimulation of the L6 spinal nerve.

Stimulation with 1 V decreased frequency of RMCs immediately after stimulation at 1 or 10 Hz. Significant decrease was observed at 3 min or 1 min after the onset of stimulation at 1 or 10 Hz, respectively (Fig. 2b, middle row). Response to stimulation at 10 Hz persisted over a long period, with significant decrease being continued over 11 min. However, response to 1 Hz-stimulation immediately started to recover, with the significant response being observed only at 3 min. Stimulation with 1 V at 0.1 Hz produced no consistent changes in RMCs during and after stimulation. Although stimulation with 1 V required to activate Aδ afferents also activates the larger diameter, fast-conducting Aβ fibers, the result that early inhibition was only observed when Aδ skin afferent fibers were additionally activated suggests the contributions of Aδ fibers to the early inhibition of RMCs. Our results indicate that the activation of Aδ afferents at 10 Hz produces early inhibition. On the other hand, low-frequency discharge (0.1 Hz) of Aδ afferents may have a late excitatory effect, which masks the late inhibition produced by the activation of Aβ fibers. Further, the intermediate frequency (1 Hz) appears to have both effects, i.e., early inhibition and late excitation.

With stimulation of 10 V, the frequency of RMCs was immediately decreased after stimulation at 1 and 10 Hz, and the significant decrease of RMCs was continued until 11 min after the onset of stimulation (Fig. 2b, lower row). Particularly at 10 Hz, consistent and strong inhibition was produced. RMCs completely stopped for >10 min in all rats tested. The contraction reappeared 17–22 min after stimulation. Changes in RMCs produced by 10 Hz stimulation were significantly different from those produced by 1 Hz stimulation (by two-way ANOVA). Stimulation with 10 V at 0.1 Hz produced no consistent changes in RMCs during and after stimulation. The results indicate that repetitive electrical activation of all fiber groups Aβ, Aδ, and C skin afferent fibers produced inhibition of RMCs, depending on stimulus frequency. Similar results were reported in cats by hind limb nerve stimulation [14]. Although stimulation with the 10 V required to activate C afferents also activates Aβ and Aδ fibers, the result that complete inhibition was only observed when skin afferent C fibers were additionally activated suggests that the activation of C afferents at 10 Hz contributes to both early and late inhibition.

Many C fibers are nociceptive fibers expressing the heat-sensing capsaicin-sensitive transient receptor protein vanilloid 1 (TRPV1) channel [16–18]. On the other hand, low-threshold mechanoreceptive C fibers do not express the TRPV1 channel; accordingly, they are unresponsive to noxious heat and capsaicin [19, 20]. TRPV1-positive cells compose 50–60 % of total dorsal root ganglion (DRG) neurons in L6-S1 (the segment innervating perineal skin) [18]. It has been suggested that low-threshold mechanoreceptive C fibers account for approximately 20–30 % of DRG neurons in L6-S1 [21].

**Fig. 3:**
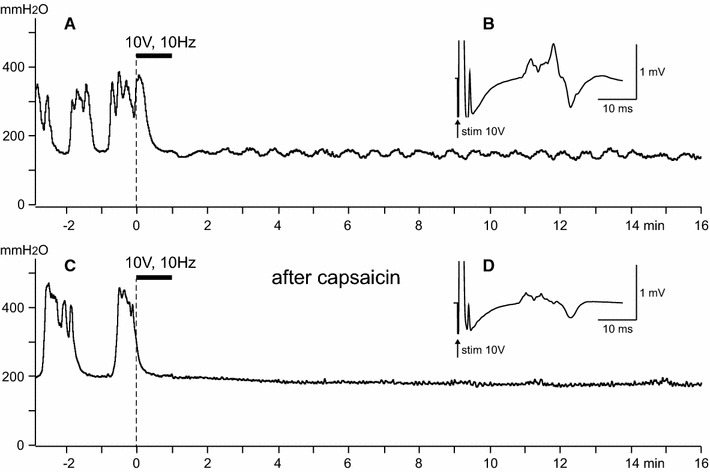
Effect of the local application of capsaicin on CBPN on the inhibition of RMCs by the electrical stimulation of CBPN. Representative results. *A*, *B* Before capsaicin treatment. *C*, *D* One hour after application of capsaicin. *A*, *C* Sample recordings of RMCs. *Horizontal heavy bars* indicate the stimulation time. *B*, *D* Compound action potentials of CBPN, averaged (600 times) during the stimulation with 10 V at 10 Hz for 1 min. The latency of the C fiber volleys in *D* is faster than in *B* probably because the nerve length may be shortened after capsaicin treatment for some reason, e.g., by movement of the stimulating or recording electrodes, or by movement of the nerve on the electrodes. The *sharp waves* with latencies of 2.5 ms (*B* and *D*) are Aδ-fiber volleys. The magnitude of that in *D* is smaller than that in *B* because there are capsaicin sensitive Aδ fibers [23, 29]

To block the conduction of TRPV1-positive C fibers, capsaicin (dissolved in 10 % Tween 80 in paraffin oil at a concentration of 1 %) was applied locally to CBPN in three rats according to the method of Petsche et al. [22]. Capsaicin treatment of CBPN for 1 h significantly reduced the amplitude of compound action potentials of the C fibers (compare Fig. 3B, D) across the application site, as reported previously [22, 23]. However, strong suppression of RMCs due to CBPN stimulation with 10 V at 10 Hz was still observed as seen before capsaicin treatment (compare Fig. 3A, C). Similar results were obtained in another two rats. Stimulation of capsaicin-treated CBPN completely stopped RMCs for 22.7 ± 6.9 (range 13–36, *n* = 3) min, which was equivalent to that in control rats without capsaicin treatment (18.7 ± 0.8 min, *n* = 6).

Petche et al. [22] showed that nociceptive C-fibers responding to strong mechanical and heat stimulation were blocked; however, unmyelinated cold fibers were not affected by capsaicin treatment. To confirm the effectiveness of conduction block of capsaicin-sensitive fibers, we examined the effect of capsaicin treatment and noxious heat-induced effect on RMCs. Noxious heat stimulation was applied to the perineal skin using a Peltier thermode as described previously [24]. Noxious heat (47 °C) stimulation for 1 min induced transient excitation during stimulation followed by a complete inhibition of bladder contractions for >10 min. Both excitatory and inhibitory effects were almost abolished by capsaicin treatment (data not shown). This result is in agreement with a report that responses of spinothalamic tract cells during heating of the cutaneous receptive field are almost eliminated by capsaicin applied to the cutaneous nerve [23]. Our result with heat stimulation suggests that TRPV1-positive fibers from the skin contribute to the changes in RMCs. Furthermore, our results with CBPN stimulation after capsaicin on CBPN suggest that TRPV1-negative skin afferent fibers, e.g., low-threshold mechanoreceptive C fibers and/or cold fibers, also contribute to the inhibition of RMCs.

In our previous study, a greater frequency of discharge was observed in unmyelinated [3.3–11.1 (7.9 ± 1.6) Hz] afferents than in myelinated [Aβ: 0.03–8.6 (2.2 ± 0.7) Hz; Aδ: 0.1–6.4 (2.9 ± 0.8) Hz] afferents during gentle rolling stimulation to a perineal skin effective in inhibiting RMCs [8] and for nocturia due to overactive bladder [25]. Taken together, the present study with electrical stimulation of CBPN, which innervates perineal skin to define the fiber groups involved in the afferent limb of the reflex inhibition of RMCs, supports the previous assumption that the activation of low-threshold mechanoreceptive afferents at an average frequency of 2 Hz for Aβ, 3 Hz for Aδ, and 8 Hz for C fibers all contribute to the inhibition of RMCs [8]. Furthermore, activity of Aβ fibers (0.1–10 Hz) contributes to delayed inhibition, activity of Aδ fibers (1–10 Hz) contributes to early inhibition, and activity of C fibers (1–10 Hz) contributes to both early and late inhibition of RMCs. Although central mechanisms of gentle rolling stimulation have partially been determined [26], mechanisms underlying the early and late inhibition are still unclear.

Stimulation of somatic nerves or somatic tissues, particularly in the sacral area, has been used for the treatment of overactive bladder [27, 28]. Our results provide an important insight for developing appropriate therapy for overactive bladder.

## References

[1] Boggs JW, Wenzel BJ, Gustafson KJ, Grill WM (2006). Frequency-dependent selection of reflexes by pudendal afferents in the cat. J Physiol.

[2] Chen ML, Shen B, Wang J, Liu H, Roppolo JR, de Groat WC, Tai C (2010). Influence of naloxone on inhibitory pudendal-to-bladder reflex in cats. Exp Neurol.

[3] Tai C, Shen B, Mally AD, Zhang F, Zhao S, Wang J, Roppolo JR, de Groat WC (2012). Inhibition of micturition reflex by activation of somatic afferents in posterior femoral cutaneous nerve. J Physiol.

[4] Xiao Z, Rogers MJ, Shen B, Wang J, Schwen Z, Roppolo JR, de Groat WC, Tai C (2014). Somatic modulation of spinal reflex bladder activity mediated by nociceptive bladder afferent nerve fibers in cats. Am J Physiol Renal Physiol.

[5] McGee MJ, Danziger ZC, Bamford JA, Grill WM (2014). A spinal GABAergic mechanism is necessary for bladder inhibition by pudendal afferent stimulation. Am J Physiol Renal Physiol.

[6] Sato A, Sato Y, Shimada F, Torigata Y (1975). Changes in vesical function produced by cutaneous stimulation in rats. Brain Res.

[7] Sato A, Sato Y, Sugimoto H, Terui N (1977). Reflex changes in the urinary bladder after mechanical and thermal stimulation of the skin at various segmental levels in cats. Neuroscience.

[8] Hotta H, Masunaga K, Miyazaki S, Watanabe N, Kasuya Y (2012). A gentle mechanical skin stimulation technique for inhibition of micturition contractions of the urinary bladder. Auton Neurosci.

[9] Woock JP, Yoo PB, Grill WM (2008). Activation and inhibition of the micturition reflex by penile afferents in the cat. Am J Physiol Regul Integr Comp Physiol.

[10] Hotta H (2016) Effectiveness and mechanism of a gentle skin stimulation for nocturia due to overactive bladder. J Physiol Sci 66(suppl 1):S23

[11] McKenna KE, Nadelhaft I (1986). The organization of the pudendal nerve in the male and female rat. J Comp Neurol.

[12] Pacheco P, Camacho MA, García LI, Hernández ME, Carrillo P, Manzo J (1997). Electrophysiological evidence for the nomenclature of the pudendal nerve and sacral plexus in the male rat. Brain Res.

[13] Sato A, Sato Y, Suzuki A (1992). Mechanism of the reflex inhibition of micturition contractions of the urinary bladder elicited by acupuncture-like stimulation in the anesthetized rats. Neurosci Res.

[14] Sato A, Sato Y, Schmidt RF (1980). Reflex bladder activity induced by electrical stimulation of hind limb somatic afferents in the cat. J Auton Nerv Syst.

[15] Su X, Nickles A, Nelson DE (2012). Neuromodulation in a rat model of the bladder micturition reflex. Am J Physiol Renal Physiol.

[16] Tominaga M, Caterina MJ, Malmberg AB, Rosen TA, Gilbert H, Skinner K, Raumann BE, Basbaum AI, Julius D (1998). The cloned capsaicin receptor integrates multiple pain-producing stimuli. Neuron.

[17] Takaishi M, Uchida K, Suzuki Y, Matsui H, Shimada T, Fujita F, Tominaga M (2016). Reciprocal effects of capsaicin and menthol on thermosensation through regulated activities of TRPV1 and TRPM8. J Physiol Sci.

[18] Kiasalari Z, Salehi I, Zhong Y, McMahon SB, Michael-Titus AT, Michael GJ (2010). Identification of perineal sensory neurons activated by innocuous heat. J Comp Neurol.

[19] Seal RP, Wang X, Guan Y, Raja SN, Woodbury CJ, Basbaum AI, Edwards RH (2009). Injury-induced mechanical hypersensitivity requires C-low threshold mechanoreceptors. Nature.

[20] Vallbo AB, Olausson H, Wessberg J (1999). Unmyelinated afferents constitute a second system coding tactile stimuli of the human hairy skin. J Neurophysiol.

[21] Li L, Rutlin M, Abraira VE, Cassidy C, Kus L, Gong S, Jankowski MP, Luo W, Heintz N, Koerber HR, Woodbury CJ, Ginty DD (2011). The functional organization of cutaneous low-threshold mechanosensory neurons. Cell.

[22] Petsche U, Fleischer E, Lembeck F, Handwerker HO (1983). The effect of capsaicin application to a peripheral nerve on impulse conduction in functionally identified afferent nerve fibres. Brain Res.

[23] Chung JM, Lee KH, Hori Y, Willis WD (1985). Effects of capsaicin applied to a peripheral nerve on the responses of primate spinothalamic tract cells. Brain Res.

[24] Watanabe N, Piché M, Hotta H (2015). Types of skin afferent fibers and spinal opioid receptors that contribute to touch-induced inhibition of heart rate changes evoked by noxious cutaneous heat stimulation. Mol Pain.

[25] Iimura K, Watanabe N, Masunaga K, Miyazaki S, Hotta H, Kim H, Hisajima T, Takahashi H, Kasuya Y (2016). Effects of a gentle, self-administered stimulation of perineal skin for nocturia in elderly women: a randomized, placebo-controlled, double-blind crossover trial. PLoS One.

[26] Hotta H, Watanabe N (2015). Gentle mechanical skin stimulation inhibits micturition contractions via the spinal opioidergic system and by decreasing both ascending and descending transmissions of the micturition reflex in the spinal cord. PLoS One.

[27] Guo ZF, Liu Y, Hu GH, Liu H, Xu YF (2014). Transcutaneous electrical nerve stimulation in the treatment of patients with poststroke urinary incontinence. Clin Interv Aging.

[28] Paik SH, Han SR, Kwon OJ, Ahn YM, Lee BC, Ahn SY (2013). Acupuncture for the treatment of urinary incontinence: a review of randomized controlled trials. Exp Ther Med.

[29] Kissin I (2008). Vanilloid-induced conduction analgesia: selective, dose-dependent, long-lasting, with a low level of potential neurotoxicity. Anesth Analg.

